# The Bog Bilberry Enigma: A Phytochemical and Ethnopharmacological Analysis of *Vaccinium uliginosum* L. Fruits in Regard to Their Alleged Toxicity

**DOI:** 10.3390/plants14172645

**Published:** 2025-08-25

**Authors:** Zuzana Vaneková, Martina Redl, Lorenz Fischer, Karin Ortmayr, Laura Jaakola, Judith M. Rollinger

**Affiliations:** 1 Division of Pharmacognosy, Department of Pharmaceutical Sciences, Faculty of Life Sciences, University of Vienna, Josef-Holaubek-Platz 2, 1090 Vienna, Austria; 2 Vienna Doctoral School of Pharmaceutical, Nutritional, and Sport Sciences, University of Vienna, Josef-Holaubek-Platz 2, 1090 Vienna, Austria; 3 Department of Arctic and Marine Biology, UiT The Arctic University of Norway, Holtvegen 62, 9016 Tromsø, Norway; 4 Division of Food Production and Society, Norwegian Institute of Bioeconomy Research (NIBIO), 1431 Ås, Norway

**Keywords:** bog bilberry, *Vaccinium uliginosum*, toxicity, cell viability, *Caenorhabditis elegans*, *Monilinia megalospora*, alcoholic fermentation

## Abstract

*Vaccinium uliginosum* (bog bilberry) is widely consumed in North America and Asia but has been historically avoided in many parts of Europe due to its alleged poisonous effects. We aimed to address this discrepancy in a systematic way with a combined phytochemical and ethnopharmacological approach, using UHPLC and UHPSFC for the chemical analysis, model organisms *Caenorhabditis elegans* and human liver cells GFP-Huh-7 for the bioactivity and toxicity testing, as well as fermentation experiments. Phytochemical analysis revealed minimal differences in the metabolite pattern between European and North American samples, with no evidence of toxic alkaloids or harmful secondary metabolites. Extracts exhibited no strongly toxic effects in the tested concentrations, neither in vitro (cell viability) nor in vivo (*C. elegans*). Berries infected by *Monilinia megalospora* showed altered flavonoid and anthocyanin contents but no increased toxicity. Notably, bog bilberries demonstrated a fermentation potential superior to *Vaccinium myrtillus,* resulting in an alcohol content of 4.8–5.8% ABV in unsweetened juices, thus potentially explaining historical accounts of inebriation. In conclusion, direct toxicity derived from these fruits is unlikely, but the alcohol content due to fruit fermentation is a plausible explanation for the folklore names (“drunk, inebriating berry”). However, additional factors such as human error, individual intolerance, or endophytic activity need to be considered.

## 1. Introduction

Bog bilberry (*Vaccinium uliginosum* L.) is a wild berry-bearing species growing in alpine, boreal, and Arctic regions of the northern hemisphere. It is a dwarf shrub with brown twigs bearing obovate to lanceolate deciduous blueish-green leaves with entire margins of a max. 35 mm long. The berries are 10 mm in diameter, have a dusty blue peel and a pale greenish-white pulp with several tiny seeds, and ripen in August–September [1,2].

Our research team has previously reviewed the inexplicable difference in attitude towards the consumption of bog bilberries in Europe vs. in other parts of the world [3]. Briefly, in Siberia, Northeast Asia, and North America, bog bilberries have always been regarded as an excellent food and medicinal source, and the berries are picked and used in preparing jams, jellies, and beverages [4,5,6]. In contrast, in the folklore knowledge of many European cultures, they are regarded as poisonous, which is reflected in the common names for the plant: “mad berry”, “drunk berry”, “inebriating berry”, and “vomit berry”. Many current books and online articles warn against ingesting the berries, claiming they can have toxic or hallucinogenic effects [7].

In the European literature, between the years 1906 and 1944, there are a handful of case reports describing toxic effects after ingesting bog bilberries; they are, however, not peer-reviewed and remain largely unverifiable. The symptoms ranged from mild (nausea, vomiting, general unwellness) to serious (changes in heart rate, disturbed vision, swallowing paralysis, hemolytic jaundice), with large differences between each case report [8,9,10,11,12,13]. The search for the postulated toxic principle of bog bilberries was occasionally attempted throughout the 20th century, but without any conclusive results [13,14,15]. The first toxicological testing of *V. uliginosum* was performed after the reports of poisoning at the eastern front of WWII. Extracts of the berries, prepared according to the Stas-Otto separation method, were tested on a living cat to observe blood pressure changes, and on an isolated dog pupil and an isolated dog intestine for parasympathomimetic effects, with no observable changes in all models and samples [13]. Piechocka and Szymczyk prepared an array of extracts aimed to concentrate alkaloids and other potentially toxic glycosides. All extracts were tested on mice (10 mice per extract, 70 in total), but none of them produced any negative effects, and the two recorded deaths were caused by catheter injury. Moreover, Piechocka herself ingested three 250 g servings of fresh bog bilberries every 30 min, 750 g in total, without any noticeable effects [14]. After the phytochemical analysis of bog bilberry leaves by Nees et al., 1973 [15], the mentions of bog bilberry toxicity faded away from the scientific literature. There is no current peer-reviewed literature that investigates the possible reasons for the avoidant attitude towards bog bilberries in some European regions.

We have previously postulated several hypotheses that could explain a potential bog bilberry toxicity [3]:

1. The parasitic fungus *Monilinia megalospora* (Woronin) Whetzel, which can infect the bog bilberry pericarp, may biosynthesize toxic secondary metabolites. So far, the metabolite profile of *Monilinia* species remains largely unexplored.
2. Another accidentally ingested plant species growing in the same habitat may have been responsible for the poisoning cases. Among others, *Atropa belladonna* L., *Lonicera nigra* L., *Andromeda polifolia* L., and *Rhododendron tomentosum* Harmaja have come into question [11,12,16,17].
3. Allergy or individual intolerance can cause unexpected reactions in some individuals [10].
4. There may be a phytochemical difference between isolated populations of bog bilberries, causing some to be toxic. Plant secondary metabolites, which most commonly cause harmful or psychoactive effects in humans, are alkaloids (e.g., scopolamine), amides (e.g., mescaline), monoterpenes (e.g., thujone), sesquiterpenes (e.g., ledol), and diterpenes (e.g., grayanotoxin) [18].
5. Some sources claim that bog bilberries ferment extraordinarily well in storage, producing alcohol which then causes the inebriation [4,5,19,20].

Based on these hypotheses, the following research questions are addressed in this study:

1. Is there any significant phytochemical difference between isolated populations of *V. uliginosum* that would substantiate the reports of toxicity? Are there any metabolites unique to the harvest sites mentioned in the historical literature?
2. How common is the infection by *Monilinia megalospora*? How do the infected “mummy berries” differ from the healthy ones in their chemical makeup? Can any potentially harmful metabolites be identified?
3. Can any viability or lifespan-reducing effects be observed on the selected bioassays upon treatment with the berry extracts? If yes, can these be correlated with the content of any secondary metabolites?
4. How does the suitability of *V. uliginosum* for alcoholic fermentation compare to other similar *Vaccinium* berries? What alcohol percentages can be achieved with or without the addition of sugar and yeast? Are these percentages high enough to justify records of inebriation?

## 2. Results

### 2.1. Chemical Profiling of the Nonpolar Bog Bilberry Extracts

As the first step in the search for a potential toxic principle of *V. uliginosum*, the freeze-dried bog bilberry fruits underwent an extraction adapted from the Stas-Otto method. With this approach that uses liquid–liquid extractions at different pH values, alkaloids and alkaloid-like substances should be extracted and concentrated. Such extracts were previously prepared from bog bilberries and tested in animal models of the berry’s toxicity [13,14], but without a detailed dereplication of constituents. As a control of a berry with and without alkaloid content, deadly nightshade berries (*Atropa belladonna* L.) and common bilberry (*Vaccinium myrtillus* L.) were used, respectively.

**Figure 1:**
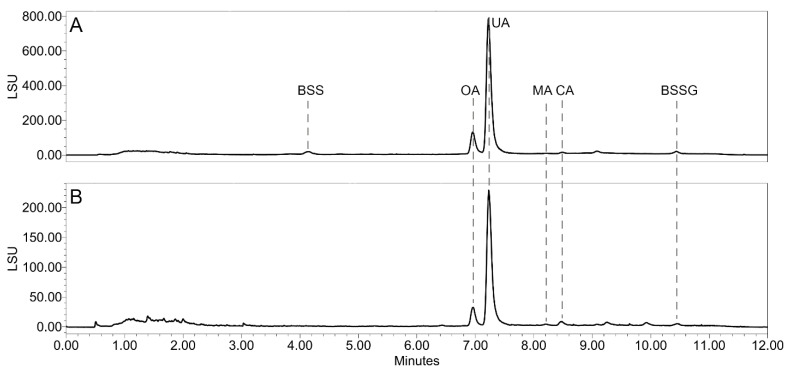
Typical UHPSFC-ELSD chromatogram of *V. uliginosum* Stas-Otto extract (**A**) and dichloromethane extract (**B**). Oleanolic acid (OA), ursolic acid (UA), maslinic acid (MA), corosolic acid (CA), β-sitosterol (BSS), and β-sitosterol glucoside (BSSG) were identified by comparison with reference compounds.

To gain first insights into the phytochemical composition of the Stas-Otto extracts, thin-layer chromatography separation was carried out. A subsequent derivatization with Dragendorff reagent as a spray reagent was negative in all *Vaccinium* samples (Supplementary Figure S1), indicating the absence of alkaloids in these samples. The Stas-Otto extracts were then analyzed by ultra-high-performance supercritical fluid chromatography coupled with UV-VIS, evaporative light scattering, and a QDa mass detector (UHPSFC-DAD-ELSD/-QDa) to gain insights into their phytochemical composition. The main constituents found in the ELSD chromatograms were characterized based on the recorded UV and MS data, compared with the literature and confirmed by reference standards. The main constituents of all *Vaccinium* Stas-Otto extracts were triterpenes oleanolic and ursolic acid, accompanied by minor amounts of phytosterols (Figure 1, detailed annotation in Supplementary Table S1).

Subsequently, defatted berry samples were extracted with dichloromethane (DCM) to achieve a targeted extraction of potentially toxic constituents of diterpene and sesquiterpene structure. As above, the UHPSFC-DAD-ELSD/-QDa analysis revealed that the major constituents of these extracts are triterpenes oleanolic and ursolic acid, as well as several other minor triterpene acids and phytosterols (Figure 1, detailed annotation in Supplementary Table S1). Overall, the DCM extracts showed a metabolite profile very similar to the Stas-Otto extracts, and no evidence of sesqui- and diterpene derivatives could be found. Identified triterpene acids and phytosterols are compounds ubiquitous in nature; they have been already described as the constituents of the berry wax [21] and are not considered harmful to human health.

### 2.2. Bioactivity Testing of LLE Berry Extracts

While the above-described extraction methods were chosen to selectively extract potential alkaloids and terpenoid substances which likely could cause harmful effects in humans, in the next step, we aimed for a more general approach. A Lead-Like-Enhanced (LLE) extraction method was selected to extract as many orally bioavailable constituents as possible, while minimizing those irrelevant for toxicity testing (such as lipids or simple sugars) or those that consistently give false positive results in bioassays, such as tannins [22]. Application of such extracts to a bioassay should therefore approximate the effects after the ingestion of the berries.

**Figure 2:**
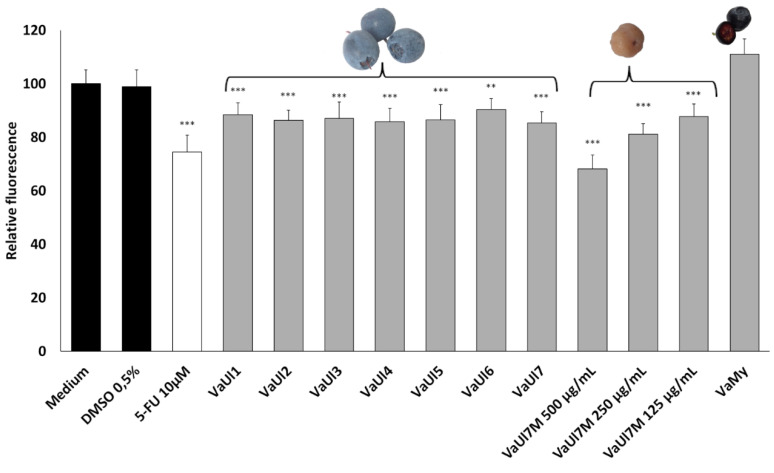
Cell viability of the Huh-7 hepatocarcinoma cells treated with LLE extracts of different berry samples at the concentration of 500 μg/mL, expressed as the relative fluorescence of a resazurin assay ± SD. Extract of the *Monilinia*-infected berries originating from Alaska was additionally tested at 250 and 125 μg/mL due to precipitation in the medium. ** *p* ≤ 0.01; *** *p* ≤ 0.001.

**Figure 3:**
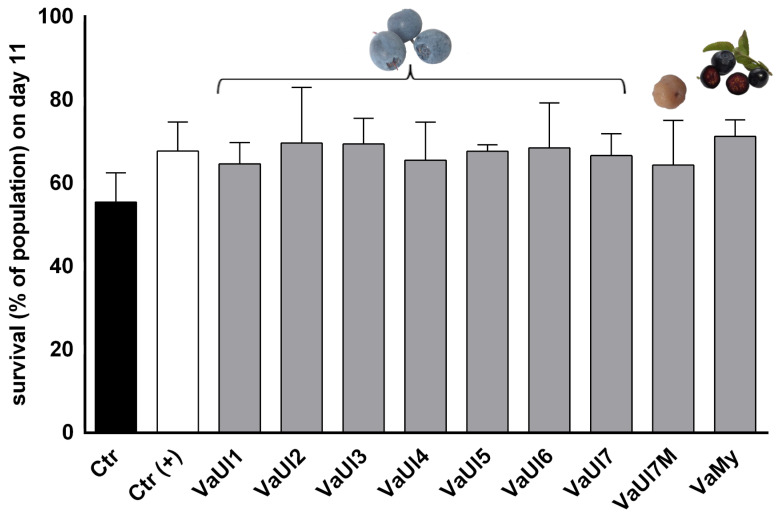
Survival of *C. elegans* populations on day 11 after treatment with LLE extracts of different berry samples at the concentration of 300 μg/mL, expressed as % of population ± SD.

LLE extracts of the berry material were tested in two different bioassays, i.e., in an in vitro cell viability assay on Huh-7 liver cells and in an in vivo survival assay in *Caenorhabditis elegans* (Figure 2 and Figure 3). The results shown here were achieved after applying relatively high concentrations of the LLE extracts to both bioassays; during the optimization step, it was apparent that concentrations in the nanogram/mL to low microgram/mL range elicit no effects in our models.

The human hepatoma cell line Huh-7 is a metabolically competent cell model to investigate hepatotoxicity in in vitro conditions [23]. In the cell viability assay, high concentrations of the LLE extracts of 500 μg/mL were necessary to elicit any effects at all. At this concentration, the bog bilberry LLE extracts showed a minor decrease in cell viability, whereas the *V. myrtillus* LLE extract caused a minor increase in the cell viability. There was no difference between the harvest locations for the bog bilberry samples. These results are consistent with the observations of Liu et al., 2010 [24], who tested an anthocyanin-rich bog bilberry extract on a hepatocellular carcinoma cell line (Hep-G2) and colorectal adenocarcinoma cell line (Caco-2), up to 1.5 mg/mL. A concentration-dependent reduction in cell viability was observed, starting at 300 μg/mL, until the cell viability reached approx. 10% at approx. 1 mg/mL.

The nematode *Caenorhabditis elegans* has developed as an emerging tool to assess a sample’s phenotypic bioactivity as well as its toxicity. A sharp decrease in lifespan of *C. elegans* is an accepted preclinical indicator for general toxicity and has been adapted for testing of natural products [25,26]. The LLE extracts were well tolerated by the N2 wild-type *C. elegans*, showing no signs of toxicity, even at the relatively high concentration of 300 μg/mL. In fact, the results indicate a slight trend towards the promotion of survival, suggesting beneficial effects in *C. elegans,* however without significance. Considering the chemical composition of the extracts, the positive impact of *V. uliginosum* and *V. myrtillus* extracts is likely attributable to their polyphenol content, particularly anthocyanins. This interpretation is consistent with previous reports showing that purified anthocyanins and anthocyanin-rich extracts exert anti-aging effects in *C. elegans* models [27,28].

### 2.3. Chemical Profiling of the LLE Berry Extracts

**Figure 4:**
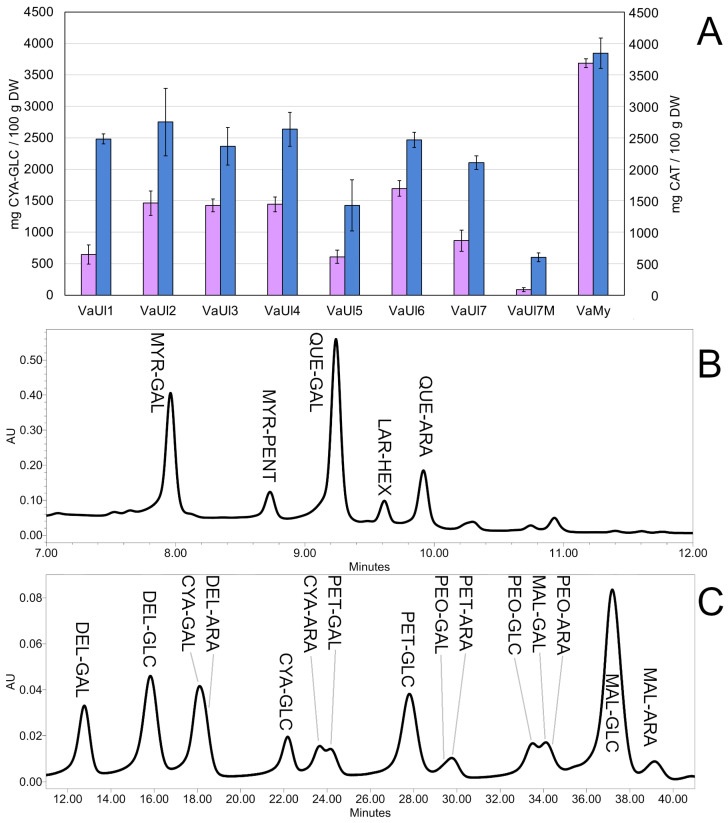
Content of total soluble polyphenols (blue) and total monomeric anthocyanins (pink) (±SD) in all tested LLE extracts (**A**); a typical UPLC-DAD chromatogram at 370 nm of a *V. uliginosum* LLE extract using System 1 (**B**); a typical UPLC-DAD chromatogram at 530 nm of a *V. uliginosum* LLE extract using System 2 (**C**).

Using spectrophotometry, we observed a high content of soluble polyphenols (TSPs) and monomeric anthocyanins (TMAs) in the LLE extracts (Table 1 and Table 2, Figure 4). With respect to VaUl samples, the highest TMA was found in the sample from Norway (VaUl6), the highest TSP in the sample from Austria (VaUl2), and the lowest content of both TSP and TMA was clearly recorded for the *Monilinia*-infected sample VaUl7M. The comparably high TMA and TSP in the *V. myrtillus* sample is well-described in the literature and is caused by anthocyanins present in the pulp as well as the peel [29].

Additionally, we used two different UHPLC-PDA-QDa methods to gain further insight into the phytochemical composition. The first method, adapted from Ancillotti et al. 2016 [29], confirmed the presence of several flavonol glycosides, which were annotated based on the glycoside [M-H]^−^ and aglycon [M-Pent-H]^−^ or [M-Hex-H]^−^ fragments under negative ionization (Supplementary Table S2) and the identity of the three highest peaks confirmed by comparison with the reference compounds: galactoside and a pentoside of myricetin, galactoside and arabinoside of quercetin, and a hexoside of laricitrin.

The second UHPLC-PDA-QDa method focused on monomeric anthocyanins revealed a wide spectrum of derivatives, out of which the dominant ones were glucosides of malvidin, delphinidin, and petunidin. The anthocyanins were dereplicated based on the glycoside [M]^+^ fragments under positive ionization and confirmed by comparison with the reference compounds (Supplementary Table S3). The elution order of anthocyanins glycosylated with the same sugar moiety was DEL < CYA < PET < PEO < MAL; regarding the retention times for the same aglycone, galactosides eluted firstly, followed by glucosides and arabinosides [29,30]. However, since it was not possible to achieve a complete baseline separation of all anthocyanins, only those with a complete baseline separation were included in Table 2.

These results are in accordance with the literature. Similar levels of secondary metabolites in *V. uliginosum* fruits were previously reported from Italy [29], Turkey [30], Lithuania [31], Finland [32,33], and China [34]. The major flavonol aglycone is quercetin followed by myricetin, with laricitrin, syringetin, and isorhamnetin in minority [33]. Out of five different anthocyanidins, the predominant ones in *V. uliginosum* are malvidin, delphinidin, and petunidin [29,33]. Our results confirm this and additionally show that galactose is the predominant sugar moiety of the flavonol glycosides, whereas glucose is the predominant sugar moiety in anthocyanins. It is apparent that the tannin removal step during the extract preparation removed the majority of the catechin and proanthocyanidin derivatives, explaining their absence in UHPLC spectra.

Differential analysis of the bog bilberry samples from different locations showed only minor variation when it comes to the ratio of these flavonoids and anthocyanins, although their total content varies. This is most likely due to the sample locations being affected by different exogenous factors, such as climate, harshness of the weather conditions, UV light exposure throughout the berry ripening season, and nutrient availability. They all have been shown to influence the contents of flavonoids and anthocyanins in *Vaccinium* berries [33,34].

### 2.4. Monilinia Megalospora Influence on the Berry Sample Bioactivity and Chemical Profile

The theory that a *Monilinia* infection is responsible for the inebriating effects of the bog bilberry was suggested by Zipf, 1944 [13], probably for the first time, and was subsequently cited by many sources (also in well-known encyclopedias [7]). Although on the first sight appearing plausible (in comparison, for example, with the infection of cereal crops with ergot fungus, *Claviceps purpurea* (Fr.) Tul.), it leaves certain issues unaddressed: many *Vaccinium* species, be it cultivated or wild, can be infected by a *Monilinia* fungal disease without causing fear of inebriation or poisoning; therefore, *V. uliginosum* and *M. megalospora* would have to possess some unique characteristics among them [3].

The sample location in Alaska was the only one where a sizeable enough sample of berries infected with the fungus *Monilinia megalospora* could be obtained; in all other European locations, the spread of the disease was rare and only a couple of infected berries could be found, if any at all. These infected berries were identified according to [35], photographed, and freeze-dried.

**Figure 5:**
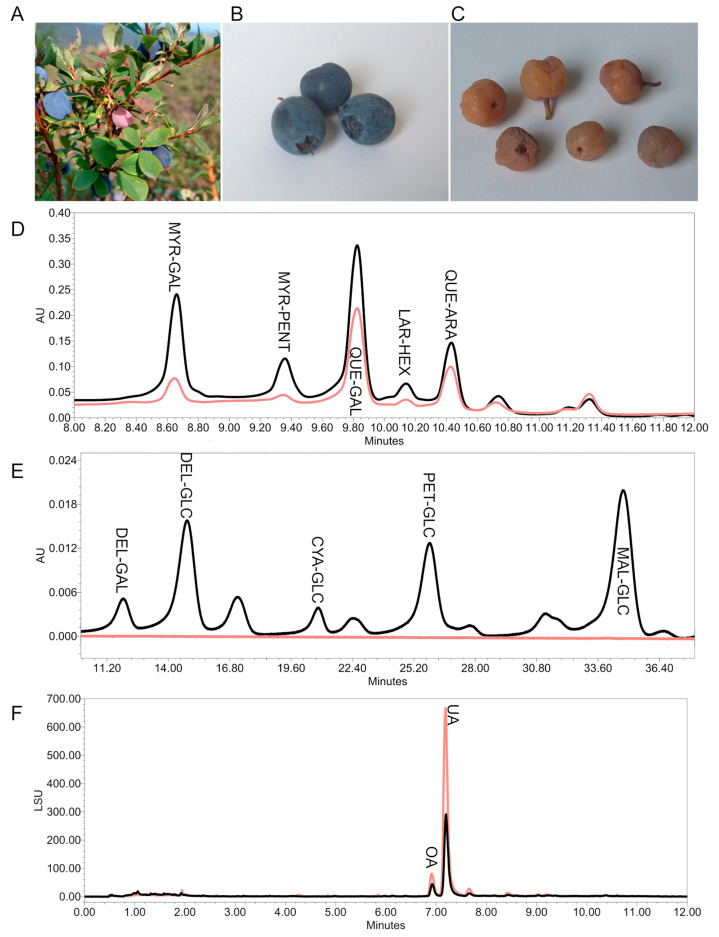
Comparison of healthy and *Monilinia megalospora*-infected berries of *V. uliginosum* from Alaska: (**A**) healthy and infected berries on the bush; (**B**) healthy berries; (**C**) infected berries; UPLC-DAD chromatograms of the LLE extracts from healthy (black) and infected (pink) berries at 370 nm using System 1 (**D**) and 530 nm using System 2 (**E**); UHPSFC-ELSD chromatograms of the DCM extracts (**F**).

As for the chemical makeup of the tested extracts, the infected berries contained higher amounts of triterpenes oleanolic and ursolic acid than the healthy sample (Figure 5). The infection by *Monilinia* seems to attenuate the synthesis of flavonoids (especially the derivatives of quercetin—myricetin and laricitrin) and almost completely stops the anthocyanin synthesis in the ripening fruits, which is reflected in the values of spectrophotometrically determined TSP and TMA, as well as in the UHPLC analysis (Table 1 and Table 2 and Figure 5). However, the dereplication of the LLE extract failed to discover compound signals unique to the *Monilinia*-infected sample. This was also the case for the DCM extracts. Overall, this study reports the effects of *Monilinia megalospora* infection on the phytochemical constitution of bog bilberry fruits for the first time.

As demonstrated in Figure 2 and Figure 3, the fungal infection does not seem to have any significant influence on the bioactivity of the LLE berry extract. Similarly, no significant difference in cell viability was found between infected and healthy *V. uliginosum* samples from Alaska, although due to the precipitation of the VaUl7M extract in the cell culture medium at 500 μg/mL, lower concentrations of 250 and 125 µg/mL had to be additionally tested.

Most of the scientific attention has been devoted to *Monilinia vaccinii-corymbosi* (J.M.Reade) Honey due to the risks it poses towards the blueberry plantations, and *M. megalospora* has been so far largely ignored. Our results suggest that the *Monilinia*-infected “mummy berries” of bog bilberry do not pose a significant threat to human health, and due to the unappetizing appearance of the *Monilinia*-infected berries, the chances of them being accidentally ingested are low. Nevertheless, the results presented here do not exclude the possibility that other endophytic organisms may be present on bog bilberries, potentially causing adverse effects after consumption.

### 2.5. Fermentation of Vaccinium Berries

Bog bilberry fruits were occasionally used to make alcoholic drinks in many parts of the world [4,20,36] and were claimed to be the reason why they are called “intoxicating berry” or “drunk berry” in so many European languages [19]. In order to address this theory, we set up several fermentation experiments. Daily weight loss monitoring was used as a measure of the fermentation process [37].

**Figure 6:**
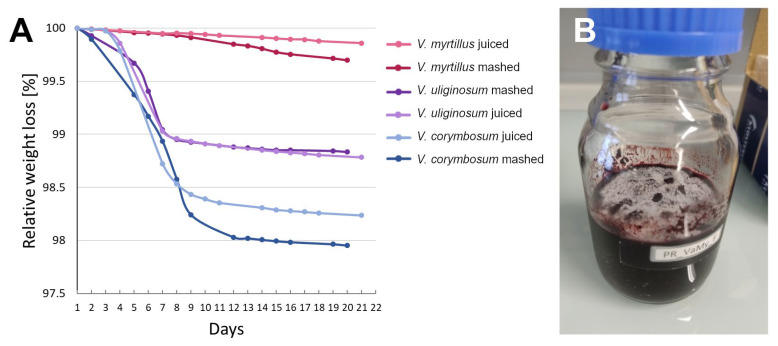
Weight loss monitoring of the fermentation process in different berry juices and purees (**A**), mold in the fermentation bottle containing mashed *V. myrtillus* on day 5 (**B**).

The first part of the fermentation experiment was set up to compare the fermenting ability of three different species of bilberry/blueberry with no addition of sugar or yeast, to test the claim of Schübeler [20], Netoliczky [4], and Jones [5] that the bog bilberries ferment better than other similar berry fruits. The results are summarized in Table 3 and Figure 6. First of all, *V. myrtillus* turned out to be not well suited for this kind of processing. Both fruit mash and juice molded instead of beginning alcoholic fermentation, despite their high Brix; the resulting liquid exhibited an unpleasant earthy aroma and signs of discoloration. In comparison, both *V. uliginosum* and *Vaccinium corymbosum* L. fermented readily and fast with no signs of mold. The resulting berry wines exhibited brilliant colors and pleasant aromas reminiscent of blueberries and redcurrants. As expected, due to the higher Brix of the raw juice, *V. corymbosum* produced a higher alcohol content than *V. uliginosum*. The monomeric anthocyanin content decreased in all samples to different degrees; TSP increased in almost all samples, the most pronounced increase being in the pureed *V. uliginosum*. These changes in metabolite content are likely due to the enzymatic activity of both fermenting microorganisms and the fruit matter itself [38].

**Figure 7:**
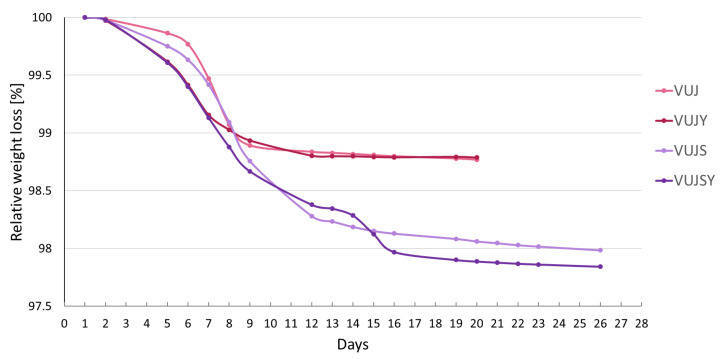
Weight loss monitoring of the fermentation process in *V. uliginosum* juice under different conditions. VUJ: raw juice, VUJY: raw juice with added yeast, VUJS: raw juice with added sugar, VUJSY: raw juice with added sugar and yeast.

In the subsequent experiment, variations in sugar content and yeast inoculation were introduced to the filtered *V. uliginosum* juice to further optimize the process. The results are summarized in Table 4 and Figure 7. A higher alcohol content was achieved in the sweetened juices, both with natural yeast and inoculated *Saccharomyces cerevisiae*. The addition of yeast resulted in an earlier onset of fermentation and a higher phenolic content in the product. The commercial yeast strain also partially attenuated the anthocyanin loss during the fermentation process. Interestingly, two-step fermentation kinetics could be observed in the VUJSY sample; the reason for this is unclear.

## 3. Discussion

We previously reviewed the inexplicable difference in the attitude towards bog bilberry in Europe vs. the rest of the world and postulated several hypotheses that might explain this strange phenomenon [3]. In this work, we approached these hypotheses using a combined phytochemical and ethnopharmacological approach.

The combined phytochemical findings from all extracts revealed only minimal differences between European and North American samples. These differences, however, do not support the theory postulated by Kupffer, 1906 [39], of isolated populations of bog bilberry being toxic. We specifically aimed to obtain samples from the places where bog bilberry toxicity was historically reported: sample VaUl1 originates in Kitzbühel, Austria, where Nevinny reported a child feeling unwell after eating bog bilberries [8]. Furthermore, sample VaUl5 is the approximate location mentioned in the bog bilberry toxicity case study by Zipf [13]. Neither of these samples stood out from the rest in their phytochemical makeup.

The Stas-Otto and dichloromethane extracts did not yield any metabolites belonging to the class of alkaloids, sesqui- or diterpenes, which are often responsible for plant toxicity. Instead, the main constituents of these extracts were triterpene acids and phytosterols [15,21,40]. A detailed isolation, identification, and determination of the major and minor triterpenes in the DCM extracts are also the topic of a separate study (Vaneková et al., 2025, in preparation).

The results of the bioactivity assays are consistent with other biological testing of *V. uliginosum* berries. One that stands out among others was performed on a cohort of 150 mice supplemented with *V. uliginosum* berry powder from Alaska, which were then subject to neurotoxic challenge with MnCl_2_. This study focused on neurotoxicity and behavior-altering effects, and the author reported no negative effects in the group with *V. uliginosum* in the feed, but without the neurotoxic treatment. So far, this study, along with the testing by Piechocka and Szymczyk [14], provides the most telling result in an animal model against the allegations of hallucinogenic or otherwise negative effects of bog bilberries [41].

Our phytochemical analysis of the bog bilberry fruits is by no means exhaustive; several other minor constituents have been discovered in the berries by other authors, such as coumarins esculetin and scopoletin [29] and iridoids monotropein, scandoside, splendoside, loganic, and deacetylasperulosidic acid [42,43]. However, so far, no discernibly toxic constituents have been discovered and the above-listed publications are in accordance with each other regarding the phytochemical make-up of bog bilberry fruits. In this context, our analysis shows a comparison of bog bilberry samples originating in countries with vastly different attitudes towards their edibility. It demonstrates a continued lack of evidence of potentially toxic constituents both in locations where the bog bilberry is avoided as well as in those where it is eaten without concern.

The parasitic fungus *Monilinia megalospora* was found to be rare, if at all present during the sample collection phase. The only sizeable enough sample originated from Alaska. Here, the infection by *Monilinia megalospora* appears to alter the content of flavonols and anthocyanins in the berries. However, no novel metabolites potentially originating in the fungal tissue in the infected “mummy berry” were found. The corresponding LLE extract did not show any significant difference to the healthy berry samples in the bioassays. Interestingly, the LLE extract derived from infected berries produced similar effects as the healthy berries from the same location despite containing, on average, only one-tenth the anthocyanin and one-third the polyphenol content found in the healthy berry extract. We propose that, in this case, the mild stimulatory effects may arise from matrix effects involving minor constituents or primary metabolites. It is noteworthy that such components may also contribute to the effects of healthy berries; however, that question was beyond the scope of this study and warrants further investigations.

There is not much known about the biosynthetic potential of *Monilinia* species infecting the berries of the *Vaccinium* genus, especially during the apothecium stage, which occurs at the same time as ripe fruits. So far, some research effort has been performed to understand the role of *Monilinia* secondary metabolites in the initial infection stage. The biosynthetic mechanisms of the initial infection of young shoots by *M. vaccinii-corymbosi* seem to be focused on the sugary matrix and volatile compounds mimicking the blueberry floral scent in order to attract insect vectors, which then facilitate the further spread of the infection [44]. The fungal hyphae also excrete phytotoxins and cell wall-degrading enzymes that cause flower and shoot blight. However, in the case of *M. vaccinii-corymbosi*, which infects highbush blueberries, the number of secondary metabolite gene clusters responsible for pathogenicity was less than half of other closely related necrotrophic fungal pathogens such as *Monilinia fruticola* (G. Winter) Honey, which causes the brown rot of stone fruits. Indeed, after the floral infection, the fungal hyphae colonize the ovary locules of the developing berry without exhibiting external symptoms of blight or rot until the berry enters the ripening phase [45]. More research into the biosynthetic potential of the *Vaccinium*-infecting *Monilinia* fungi during the apothecium stage is necessary to further elucidate this topic. Ultimately, the unappetizing appearance of the infected berries means they are unlikely to be ingested. The intoxication by *Monilinia megalospora,* therefore, appears improbable.

Based on our fermentation experiments, the hypothesis of bog bilberries being used for alcoholic beverages seems to be the most plausible one. The alcohol content of the resulting berry wine varied depending on the starting sugar concentration, but even with raw unsweetened bog bilberry juice or puree, it could reach around 5% ABV. The various local names referring to “drunk, inebriating, mad berries” may refer to the alcoholic beverages made from them [3]. This supports the theory of several authors that the alleged bog bilberry toxicity stems from the usage of these berries to produce alcoholic beverages. The Norwegian botanist Frederik Schübeler, when talking about the traditional uses of bog bilberries in Norway, states that the juice pressed from them was mixed with sugar and fermented to create wine in the Norwegian countryside, a practice for which the bog bilberry is much better suited than the common bilberry. He claims to have tested the fermentation of both kinds himself [20] and his reports align well with the traditional uses of Alaska native tribes, who stored the berries in large wooden barrels submerged in their own juice and covered with clean leaves or a layer of tallow. As soon as the berries started to ferment, they had to be stirred down once or twice per day; otherwise, the barrel would overflow. Fermented berries were then allowed to freeze and were thus conserved for the winter. Moreover, bog bilberries were regarded as the most powerful fermenting agent out of all commonly consumed plants in Alaska and were used for pickling and preserving of other foods [5].

It remains unclear why the raw *V. myrtillus* puree and juice was so unsuitable for alcoholic fermentation in our experimental setup. There are a few studies optimizing the process of making alcoholic beverages from *V. myrtillus,* and they usually do not use the raw juice, but instead dilute it with water 1:1, pasteurize it, and add back sugar to reach the desired °Brix [37,46,47], or add sugar, yeast, and nutritional supplement to the raw juice [38]. From the available literature, three reasons appear plausible:

1. It is possible that the berries are not sufficiently populated by wild yeast strains to enable successful alcoholic fermentation, and therefore require inoculation.
2. According to Litwiller’s experiments on *Vaccinium ovatum* Pursh juice, the lack of available nitrogen might also play a role in a berry’s resistance to fermentation [48]. In modern brewing technologies, this is remediated by adding nutritional supplements [38].
3. The practice of diluting the raw juice would also suggest that *V. myrtillus* contains metabolites that inhibit bacterial and yeast growth, but not mold. This is the case with cranberries *Vaccinium macrocarpon* Aiton and *Vaccinium oxycoccos* L. and lingonberries *Vaccinium vitis-idaea* L., which contain high concentrations of benzoic acid and therefore keep extraordinarily well without any additional conservation [48,49]. A quantitative analysis with GC-MS by Klavins and colleagues compared levels of benzoic acid in several berry species: *V. corymbosum* 0.64 µg/g, *V. uliginosum* 0.51 µg/g, and *V. myrtillus* 6.13 µg/g of fresh berries. The levels of benzoic acid in *V. myrtillus* were not as high as in cranberry or lingonberry (37.08 and 164.40 µg/g, respectively) [50], but nevertheless were much higher than in the other two species compared in the present work.

As some authors previously suggested, a few cases of negative reactions after ingesting bog bilberries were likely caused by individual intolerance [10,14,19,39], or by mistakenly ingesting a different poisonous plant [12,16].

Ultimately, the allegations of bog bilberry toxicity appear to be unsubstantiated. The present work joins the existing body of evidence against the allegations of bog bilberry toxicity.

## 4. Materials and Methods

### 4.1. Plant Material

Fully ripe *V. uliginosum* (abbreviated VaUl) berries were collected in various locations in Europe and North America between 2020 and 2023 (Table 5), one batch per location (batch size depending on the berry availability). The berries were frozen within 24 h after picking and stored frozen until analysis. From each sample, 100 g of frozen fruits were homogenized, freeze-dried, and ground. Berry powder was then briefly kept in a desiccator at room temperature and labeled VaUl. Common bilberry *V. myrtillus* L. fruits originating from Slovakia and purchased from a local vendor, and blueberry (most likely *V. corymbosum* L.) fruits originating from Austria were both treated the same as *V. uliginosum* and labeled VaMy and VaCo, respectively.

Deadly nightshade (*Atropa belladonna* L.) berries were collected in the medicinal garden of the Faculty of Life Sciences, University of Vienna, homogenized, freeze-dried, and ground. Berry powder was kept in a desiccator at room temperature until analysis and labeled AtBe.

Voucher specimens from all samples are stored at the Division of Pharmacognosy, University of Vienna.

### 4.2. Chemicals

Reference standards were supplied as follows: cyanidin-3-*O*-glucoside (CYA-GLC), delphinidin-3-*O*-glucoside (DEL-GLC), malvidin-3-*O*-glucoside (MAL-GLC), peonidin-3-*O*-glucoside (PEO-GLC), (+)-catechin (CAT), quercetin-3-*O*-galactoside (QUE-GAL), quercetin-3-*O*-arabinoside (QUE-ARA), myricetin-3-*O*-galactoside (MYR-GAL), atropine (ATR), oleanolic acid (OA), ursolic acid (UA), maslinic acid (MA), corosolic acid (CA), β-sitosterol (BSS), and β-sitosterol glucoside (BSSG) were purchased from Sigma–Aldrich, St. Louis, MO, USA.

CC-6 polyamide for column chromatography was purchased from Carl Roth GmbH, Karlsruhe, Germany. All solvents were of HPLC grade and purchased from VWR Chemicals, Vienna, Austria. Compressed 4.5-grade CO_2_ with a purity of 99.995% (purchased from Messer SE & Co. KGaA, Bad Soden, Germany) was used. Ultrapure water was taken from a Milli-Q system supplied by Millipore (Billerica, MA, USA). Formic acid, Folin–Ciocalteu reagent, sodium carbonate, glacial acetic acid, hydrochloric acid (36–37%), potassium chloride, and sodium acetate trihydrate were supplied by Merck (Darmstadt, Germany).

### 4.3. Extraction Methods

In order to test the potential content of alkaloids in the *V. uliginosum* berries, an adapted Stas-Otto method [13] was used. We defatted 2 g of freeze-dried and ground berry material from each of the locations with 10 mL n-hexane and dried it again under a soft stream of air. The plant material was then pre-treated with 1 mL of concentrated ammonia to liberate the alkaloid base. Afterwards, the powder was extracted with 2 × 15 mL CHCl_3_ in an ultrasonic bath for 15 min, then shaken for 15 min and filtered. The filter was washed with CHCl_3_, the extract evaporated on rotary evaporator completely, and the residue in the round flask dissolved in 15 mL 0.1 N H_2_SO_4_. The aqueous extract was transferred to a separatory funnel, neutralized with concentrated ammonia to pH 9–10, and shaken with 2 × 10 mL chloroform. Chloroform fractions were combined and dried on a rotary evaporator, with the final amount transferred to a pre-weighed labeled vial.

For the preparation of dichloromethane (DCM) extracts, 1 g of freeze-dried, ground, and defatted berry material from each of the locations was placed into a centrifuge tube, respectively, and then mixed with 10 mL of DCM, placed into an ultrasonic bath for 15 min, and then centrifuged for 10 min at 3500 rpm. The supernatant was removed afterwards and filtered through cotton wool into a sample tube. The material was extracted 3 times in this manner, and the DCM extracts were collected all together, filtered through filter paper if necessary, and dried on rotary evaporator, with the final amount transferred to a pre-weighed labeled vial.

Combined dichloromethane-methanolic extraction of the berries was adapted from the Lead-Like Enhanced (LLE) extraction protocol [22]. Briefly, 0.6 g of berry powder was defatted twice with 5 mL hexane, then extracted subsequently with 7 mL dichloromethane (DCM) and 13 mL methanol (MeOH) in an ultrasound bath for 15 min. DCM and MeOH extracts were combined, dried, reconstituted in MeOH, loaded onto an SPE cartridge filled with CC-6 polyamide gel, and washed with 8 mL MeOH. The resulting MeOH eluate was concentrated on a rotary evaporator and freeze-dried [22].

Extraction yields for all extracts can be found in Supplementary Table S4.

### 4.4. Determination of Anthocyanin Content

Total monomeric anthocyanins (TMAs) were determined with the pH differential method [51] using cyanidine-3-*O*-glucoside as the reference standard, with few modifications. Aliquots of 100–200 µL of berry extract were diluted in buffer solutions at pH = 1 and pH = 4.5 to obtain a final volume of 10 mL, in triplicate for each sample. The absorbance (Abs) of both solutions were measured three times at 520 and 700 nm, and the quantity “ΔAbs” was calculated according to the following equation:

$$∆\mathrm{Abs}\;=\;\left({\mathrm{Abs}}_{\mathrm{pH}=1}^{520\mathrm{nm}}\;-{\;\mathrm{Abs}}_{\mathrm{pH}=1}^{700\mathrm{nm}}\right)-\left({\mathrm{Abs}}_{\mathrm{pH}=4.5}^{520\mathrm{nm}}-{\mathrm{Abs}}_{\mathrm{pH}=4.5}^{700\mathrm{nm}}\right)$$

Similarly, ΔAbs values were calculated for different concentrations of CYA-GLC reference standard and plotted as a function of corresponding CYA-GLC concentrations. The results were expressed as milligrams of cyanidin-3-*O*-glucoside/100 g of dry weight (DW) berry powder [29].

### 4.5. Determination of Total Soluble Polyphenols

Total soluble polyphenols (TSPs) were spectrophotometrically determined at λ_EX_ = 740 nm according to the Folin–Ciocalteau method using (+)-catechin as a reference standard [52]. Then, 100–200 μL of the extract (depending on the polyphenol concentration in the extract, in triplicate for each sample) was mixed with 200 μL of Folin–Ciocalteau reagent. After 3 min, 400 μL of an aqueous solution saturated with sodium carbonate was added, and the mixture obtained was made up to 10 mL with ultrapure water. The solution was dark incubated for 1 h; afterwards, the absorbance was measured thrice at 740 nm and the polyphenol concentration calculated according to a catechin calibration curve; accordingly, the results were expressed as milligrams of catechin/100 g DW [29].

### 4.6. UPLC-DAD-MS

To analyze the secondary metabolites of the LLE extracts, a Waters (Milford, MA, USA) Acquity UPLC instrument consisting of a sample-, quaternary solvent- (QSM), isocratic solvent- (ISM), and column manager as well as a photodiode array (PDA) and Quadrupole Dalton (QDa) detector was used. Nitrogen served as the nebulizing gas for QDa operation. The operating software was Empower 3.

The settings for the analytical instruments were as follows:

- Concentration of samples: 15 mg/mL for extracts, 1 mg/mL for standard compounds; dissolved in MeOH. Injection volume: 10 µL for extracts, 1 µL for standards.
- 90% H_2_O + 10% MeOH with 10 mM ammonium formate as ISM solvent, used for an appropriate dilution of the eluate before entering the mass spectrometer.
- Flow rate: 0.3 mL/min for QSM, 0.4 mL/min for ISM.
- Column: Acquity BEH C18, 2.1 × 100 mm, 1.7 μm.
- PDA detector settings: detection wavelength 370 nm for flavonoids, 530 nm for anthocyanins.
- QDa detector settings: capillary voltage 0.8 kV (positive and negative), cone voltages 15 V (positive) and 30 V (negative), mass range 100–1000 *m*/*z*, probe temperature 600 °C.

Two different mobile-phase systems were used, adapted from [29]:

- System 1—column temperature: 45 °C, mobile phase: A: water + 0.1% formic acid, B: acetonitrile + 0.1% formic acid, C: methanol + 0.1% formic acid. Gradient: Initial: A 98.0%, B 0.0%, C 2.0%; 1.0 min: A 98.0%, B 0.0%, C 2.0%; 2.0 min: A 85.0%, B 0.0%, C 15.0%; 15.0 min: A 40.0%, B 0.0%, C 60.0%; 16.0 min: A 5.0%, B 0.0%, C 95.0%; 18.0 min: A 2.0%, B 98.0%, C 0.0%; 22.0 min: A 2.0%, B 98.0%, C 0.0%; 22.5 min: A 98.0%, B 0.0%, C 2.0%; 24.0 min: A 98.0%, B 0.0%, C 2.0%.
- System 2—column temperature: 50 °C, mobile phase: A: water + 1% formic acid, B: methanol + 1% formic acid. Gradient: Initial: A 93.0%, B 7.0%; 43.0 min: A 78.0%, B 22.0%; 43.5 min: A 5.0%, B 95.0%; 48.0 min: A 5.0%, B 95.0%; 48.5 min: A 93.0%, B 7.0%; 50.0 min: A 93.0%, B 7.0%.

### 4.7. UHPSFC-DAD-ELSD-MS

To analyze nonpolar constituents of the Stas-Otto and DCM extracts, a Waters Acquity UPC^2^ instrument consisting of a convergence-, sample-, binary solvent-, isocratic solvent-, and column manager as well as a photodiode array (PDA), evaporative light scattering (ELSD) and Quadrupole Dalton (QDa) detector was used. Nitrogen served as the nebulizing gas for QDa and ELSD operation. The operating software was Empower 3.

The settings for the analytical instruments were as follows:

- Concentration of samples: 4 mg/mL for extracts, 1 mg/mL for standard compounds; dissolved in 4:6 mixture of hexane and isopropanol. Injection volume: 4 µL.
- Mobile phase: supercritical CO_2_ as A, methanol as B.
- 95% MeOH + 5% H_2_O with 10 mM ammonium formate as ISM solvent, used for an appropriate dilution of the eluate before entering the mass spectrometer.
- Flow rate: 1 mL/min for both BSM and ISM. Backpressure: 2000 psi.
- Gradient: Initial: A 95.0%, B 5.0%; 3.0 min: A 90.0%, B 10.0%; 10.5 min: A 74.0%, B 26.0%; 11.0 min: A 95.0%, B 5.0%; 12.0 min: A 95.0%, B 5.0%.
- Column: Torus 1-AA (1-Aminoanthracene), 130 Å, 1.7 µm, 3 × 100 mm.
- Column temperature: 50 °C.
- PDA detector settings: detection wavelength 210 nm.
- ELS detector settings: detector gain 100, gas pressure 40.0 psi, nebulizer in heating mode, drift tube temperature 50.0 °C.
- QDa detector settings: capillary voltage 0.8 kV (positive and negative), cone voltages 15 V (positive) and 30 V (negative), mass range 100–1000 *m*/*z*, probe temperature 600 °C.

### 4.8. Cell Viability Assay

Cell viability was assessed in Huh-7 hepatocarcinoma cells using a resazurin assay. Upon addition to the cell culture medium, resazurin is reduced by the metabolic activity of viable cells (i.e., by reducing agents like NADH), which generates the fluorescent product resorufin. The amount of the transformation product is then quantified by fluorescence measurement with an excitation wavelength of 535 nm and emission wavelength of 590 nm. Huh-7 cells were originally obtained as a gift to K.O. from P. Blattmann (Institute of Molecular Systems Biology, ETH Zurich, Switzerland). The cell line is listed in the Japanese Cancer Research Resources Bank (JCRB) with the catalog number JCRB0403. Cells have been maintained in the lab in RPMI-1640 cell culture medium (Sigma-Aldrich, cat. no. R8758) with 5% dialyzed fetal bovine serum (Fisher Scientific, Waltham, MA, USA, cat. no. 10594553) and 1% penicillin–streptomycin (Sigma-Aldrich, cat. no. P4333). Approximately 24 h prior to the addition of extracts, the cells were seeded in 96-well plates (Fisher Scientific, cat. no. 10212811) at a cell density of 2 × 10^4^ cells/mL with 150 µL cell suspension per well. Cell cultures were incubated overnight at 37 °C in 5% CO_2_ atmosphere for cell attachment and equilibration. To test the effect of different bog bilberry extracts on the viability of Huh-7 cells, the different extracts were added to individual wells in triplicate at a final concentration of 500 µg/mL. Sample VaUl7M precipitated when added to the cell culture medium. Therefore, we additionally tested this extract at lower concentrations of 250 and 125 µg/mL. Separate cell populations were treated with 0.5% DMSO (vehicle control, Sigma-Aldrich, cat. no. D2650) or 10 µM 5-fluorouracil (Sigma-Aldrich, cat. no. F6627) as negative and positive controls, respectively. At 96 h after treatment with the different extracts, resazurin (Sigma-Aldrich, cat. no. R7017) was added to the culture supernatants at a final concentration of 10 µg/mL. The cultures were incubated for 2 h at 37 °C in a 5% CO_2_ atmosphere, before fluorescence was measured in each well using a TECAN Spark Cyto plate reader (Tecan Group Ltd., Männedorf, Switzerland). Cell viability (%) was calculated as the ratio of relative fluorescence of the sample to the relative fluorescence of the untreated control multiplied by 100. Statistical significance was determined by a t-test, with differences considered significant at *p* ≤ 0.05.

### 4.9. Caenorhabditis Elegans Survival Assay

*C. elegans* wild-type N2, var. Bristol and *Escherichia coli* OP50, used for the survival experiments, were obtained from the Caenorhabditis Genetics Center, University of Minnesota. Media and NGM (nematode growth medium) plates were prepared as described by [53]. *E. coli* OP50 cultures and *C. elegans* were maintained following the protocol outlined by [25]. OP50 *E. coli* was cultured in LB medium for 8 h at 37 °C, collected by centrifugation, washed twice with double-distilled water, and finally air-dried. The bacterial pellet was resuspended in S-complete medium at a concentration of 100 mg/mL and stored at 4 °C until use. *C. elegans* wild-type N2, var. Bristol were maintained on NGM agar plates, seeded with OP50, at 16 °C. Worms were monitored routinely and transferred weekly to fresh OP50-seeded plates. For the survival assay, worms were transferred to fresh NGM plates, three days before synchronization. During the experiment, 96-well plates were stored at 25 °C. The survival assay was performed according to the method described in [26]. For the synchronization of the worm culture, N2 *C. elegans* were harvested from the NGM plates and suspended in ddH_2_O. The worms were exposed to an alkaline hypochlorite solution (bleaching solution) for 5–10 min to lyse the worms’ cuticles and to release the eggs. The lysis was stopped by adding M9 buffer. The eggs were collected by centrifugation at 2500× *g* and washed twice with M9 buffer and once with S-complete medium. The eggs were stored in S-complete at room temperature on a mortar for 48 h until the larvae hatched.

An amount of 5–20 L1 larvae were dispensed into each of the inner wells of three 96-well plates; the outer wells were filled with S-complete medium and served as a diffusion barrier. The worms were fed with fresh OP50 *E. coli* (6 mg/mL) and stored at 25 °C. After 24 h, worms were sterilized with 5-fluorodeoxyuridine (0.12 mM final; Sigma-Aldrich). Young adult worms were treated with the samples, the positive control 100 µM cyanidin chloride, and the vehicle control, 0.7% DMSO. All conditions were tested in triplicate wells and the experiment was conducted in three parallel replicates. On the fourth day of adulthood, animals were fed with 3.8 µL OP50 solution (100 mg/mL) to prevent starvation. On the 11th day of the experiment, the worm survival was evaluated under the dissecting microscope. Worms were considered dead if they exhibited no response to mechanical stimulation or light exposure.

Survival rates were expressed as the percentage of live worms relative to the total population per condition (mean survival rate) ± SD. Data were visualized as bar charts using GraphPad Prism 9. Statistical significance was determined by one-way ANOVA followed by Dunnett’s post hoc test, with differences considered significant at *p* ≤ 0.05.

### 4.10. Fermentation

To test the alcohol hypothesis, several fermentation experiments were set up in sterilized 250 mL glass bottles equipped with S-shaped fermentation airlocks. Each fermentation bottle was filled with 150 mL sample. Frozen fruits from *V. uliginosum* (pooled from multiple locations in Austrian Alps), *V. corymbosum*, and *V. myrtillus* were used, processed as follows:

- Whole frozen fruits, roughly mashed with no further processing, one bottle per species.
- Whole frozen fruits, mashed and juiced through a cheesecloth, one bottle per species.
  Subsequently, frozen fruits of *V. uliginosum* were pureed, filtered through a 0.5 mm sieve, and divided between four sets of conditions, two bottles each:
- 150 mL raw juice with no additions, labeled VUJ_1 and VUJ_2.
- 150 mL raw juice with 10 g added sugar, labeled VUJS_1 and VUJS_2.
- 150 mL raw juice with 0.125 g added brewer’s yeast (*Saccharomyces cerevisiae* Oenoferm^®^ X-thiol, Erbslöh Geisenheim AG, Geisenheim, Germany), labeled VUJY_1 and VUJY_2.
- 150 mL raw juice with 10 g added sugar and 0.125 g brewer’s yeast, labeled VUJSY_1 and VUJSY_2.

All fermentation bottles were kept at room temperature (approx. 22 °C), monitored daily for temperature and the presence of bubbles or mold, stirred once per day, and weighed. The fermentation was allowed to proceed freely for at least two weeks with no further additions. The loss of weight due to the escaping CO_2_ was used to estimate the fermentation kinetics. Fermentation was assumed complete when the weight remained constant (i.e., <0.1 g weight loss) at two consecutive monitoring time points [37].

After fermentation, all samples were centrifuged at 3600× *g* for 5 min, and the supernatants were kept at −20 °C until analysis.

- The same set of analyses was applied to all samples before and after fermentation.
- The pH values were measured with a pH meter (HI223, Hanna Instruments, Germany).
- The °Brix values were measured with a handheld Brix meter (RHB-32ATC, Hong Han GmbH, Germany).
- The alcohol content was determined by the water vapor distillation method adapted from *European Pharmacopoeia, 5th ed.* [54], and expressed as alcohol by volume (ABV).

The total anthocyanin and phenolic content was determined as described above, expressed as mg standard equivalents per mL.

## Figures and Tables

**Table 1:** Total soluble polyphenols (TSPs) and the ratios of the individual flavonoid glycosides based on the UHPLC-DAD analysis at 370 nm in all samples.

Sample	TSP [mg CAT/100 g DW] ± SD	% of the Total Peak Area in a UHPLC-DAD Chromatogram at 370 nm				
		MYR-GAL *	MYR-PENT	QUE-GAL *	LAR-HEX	QUE-ARA *
VaUl1	2481.67 ± 81.10	23.77	4.93	40.41	4.99	9.88
VaUl2	2752.08 ± 537.12	23.74	4.30	42.89	4.24	11.47
VaUl3	2365.47 ± 297.73	17.58	5.59	46.24	5.47	10.50
VaUl4	2636.52 ± 271.80	22.90	5.18	43.61	4.34	10.66
VaUl5	1424.59 ± 404.73	20.62	5.93	42.77	6.51	10.15
VaUl6	2468.18 ± 120.70	25.80	5.36	37.88	4.32	11.09
VaUl7	2103.39 ± 107.23	23.17	9.12	34.22	3.88	13.27
VaUl7M	600.13 ± 72.01	9.87	2.85	38.47	2.22	16.59
VaMy	3843.57 ± 242.50	7.15	n.d.	34.69	n.d.	5.12

* Identity confirmed by reference compounds. n.d.—not detected.

**Table 2:** Total monomeric anthocyanins (TMAs) and the ratios of the individual anthocyanin glycosides based on the UHPLC-DAD analysis at 530 nm in all samples.

Sample	TMA [mg CYA-GLC/100 g DW] ± SD	% of the Total Peak Area in a UHPLC-DAD Chromatogram at 530 nm					
		DEL-GAL	DEL-GLC *	CYA-GLC *	PET-GLC *	MAL-GLC *	MAL-ARA
VaUl1	646.93 ± 153.16	3.94	12.93	3.05	13.48	30.52	4.56
VaUl2	1461.70 ± 193.73	8.98	10.95	2.87	10.34	31.20	4.13
VaUl3	1426.07 ± 102.27	6.23	10.48	2.28	11.17	30.91	5.28
VaUl4	1442.22 ± 119.67	5.74	11.06	2.12	11.52	30.84	6.08
VaUl5	609.40 ± 106.71	6.77	12.66	2.87	13.14	33.45	3.25
VaUl6	1694.76 ± 124.79	8.54	14.16	4.43	12.36	29.22	2.24
VaUl7	864.23 ± 170.54	6.27	21.06	4.32	18.11	30.91	1.20
VaUl7M	85.61 ± 33.10	n.d.	n.d.	n.d.	n.d.	n.d.	n.d.
VaMy	3684.15 ± 70.67	12.79	13.49	10.42	10.19	9.38	1.77

* Identity confirmed by reference compounds. n.d.—not detected.

**Table 3:** Physicochemical indexes of the different blueberry/bilberry species before and after fermentation.

Species	Sample n = 1	pH	°Brix	ABV [%] ± SD	TSP [mg CAT/mL] ± SD	TMA [mg CYA-GLC/mL] ± SD
*V. uliginosum*	before	2.93	8	-	3054.07 ± 130.66	628.49 ± 23.28
	mashed	2.91	5	4.8 ± 0.4	5035.25 ± 86.40	415.49 ± 23.67
	juiced	2.96	5	5.8 ± 0.2	2922.82 ± 84.55	460.97 ± 34.94
*V. corymbosum*	before	2.91	14	-	269.78 ± 1.02	55.56 ± 13.54
	mashed	2.97	4.8	13.2 ± 0.4	809.08 ± 36.20	74.43 ± 10.95
	juiced	2.92	4.2	10.4 ± 0.8	788.40 ± 8.20	63.17 ± 10.75
*V. myrtillus*	before	2.96	10	-	3305.37 ± 15.78	1280.76 ± 63.46
	mashed	2.83	9	1.75 ± 0.25	3882.62 ± 64.68	450.03 ± 83.17
	juiced	2.95	10.2	2.6 ± 0.6	3592.13 ± 205.16	960.32 ± 75.46

**Table 4:** Physicochemical indexes of the bog bilberry wines fermented under different conditions: VUJ: raw juice, VUJS: raw juice with added sugar, VUJY: raw juice with added yeast, VUJSY: raw juice with added sugar and yeast.

Sample n = 2	pH	°Brix	ABV [%] ± SD	TSP [mg CAT/mL] ± SD	TMA [mg CYA-GLC/mL] ± SD
before	2.93	8 (VUJ and VUJY) 16 (VUJS and VUJSY)	-	3054.07 ± 130.66	628.49 ± 23.28
VUJ	2.84	5	5.2 ± 0.28	2692.98 ± 136.57	419.24 ± 29.14
VUJS	2.86	9.2	8.2 ± 0.2	3522.15 ± 308.59	447.41 ± 44.44
VUJY	2.81	4.8	7.3 ± 2.83	3219.84 ± 172.98	472.75 ± 38.70
VUJSY	2.95	8.2	8.2 ± 1.82	3611.41 ± 270.35	597.30 ± 145.88

**Table 5:** List of *V. uliginosum* berry material and sample locations.

	Site Characteristics						Collection Date	Voucher Specimen
Abbr.	Country	GPS Coordinates	Site Name	Elevation [m a.s.l.]	Biotope	Relevant Plants Present		
VaUl1 ^1^	Austria	N 47.4591 E 12.3709	Schwarzsee, Kitzbühel	780	Peat bog by an alpine lake	*Andromeda polifolia* L.	08.09.2020	BBE_048
VaUl2	Austria	N 47.2933 E 11.7618	Kleiner Gamsstein	1815	Alpine blanket bog	-	11.08.2022	BBE_058
VaUl3	Slovakia	N 49.4294 E 19.4996	Klin bog	610	Peat bog	*A. polifolia*, *Rhododendron tomentosum*	02.09.2020	BBE_006
VaUl4	Czechia	N 48.8539 E 14.8352	Kočičí bláto	460	Dried peat bog	*R. tomentosum*	23.08.2020	BBE_036
VaUl5 ^2^	Estonia	N 58.2564 E 27.0559	Võnnu	50	Clearing in pine forest	-	16.07.2023	BBE_158
VaUl6	Norway	N 69.6332 E 18.9950	Fløya, Tromsø	450	Tundra heath	-	11.09.2023	BBE_096
VaUl7	Alaska, USA	N 69.9906 W 147.7928	Old Murphy Dome Road	530	Clearing in spruce forest	*R. tomentosum*	27.07.2023	BBE_088
VaUl7M ^3^	Alaska, USA	N 69.9906 W 147.7928	Old Murphy Dome Road	530	Clearing in spruce forest	*R. tomentosum*	27.07.2023	BBE_088

^1^ This is the location mentioned in the bog bilberry toxicity case report by Nevinny, 1908 [8]. ^2^ This is the approximate location mentioned in the bog bilberry toxicity case study mentioned by Zipf, 1944 [13]. ^3^ This sample consists of *V. uliginosum* berries infected with *Monilinia megalospora* fungus.

## Data Availability

Raw data will be made available upon request.

## Supplementary Materials

The following supporting information can be downloaded at: https://www.mdpi.com/article/10.3390/plants14172645/s1, Figure S1: TLC analysis of all Stas-Otto extracts; Figure S2: UHPSFC-ELSD traces of all Stas-Otto extracts; Figure S3: UHPSFC-ELSD traces of all DCM extracts; Figure S4: UHPLC-PDA traces of all LLE extracts at 370 nm using System 1; Figure S5: UHPLC-PDA traces of all LLE extracts at 530 nm using System 2; Figure S6: Confluence endpoints of the cell viability assay; Figure S7: Cell confluence monitoring, showing growth curves for the cells treated with the extracts, compared to the medium and DMSO control, as well as the 5-FU-treated cells; Table S1: MS annotation of nonpolar extract constituents in the UHPSFC system; Table S2: MS annotation of LLE extract constituents in the UHPLC system 1; Table S3: MS annotation of LLE extract constituents in the UHPLC system 2; Table S4: extraction yields for the three methods used in the study.
