# Supplementary material for: The Bog Bilberry Enigma: A Phytochemical and Ethnopharmacological Analysis of Vaccinium uliginosum L. Fruits in Regard to Their Alleged Toxicity
Source: Plants (Basel). 2025 Aug 25;14(17):2645. doi: 10.3390/plants14172645 (PMC12430337; doi:10.3390/plants14172645)
Supplement: Supplementary file 1 [file plants-14-02645-s001.zip › plants-3807778-supplementary.pdf]

The Bog Bilberry Enigma: a phytochemical and ethnopharmacological analysis of *Vaccinium uliginosum* L. fruits in regard to their alleged toxicity – Supplementary Material

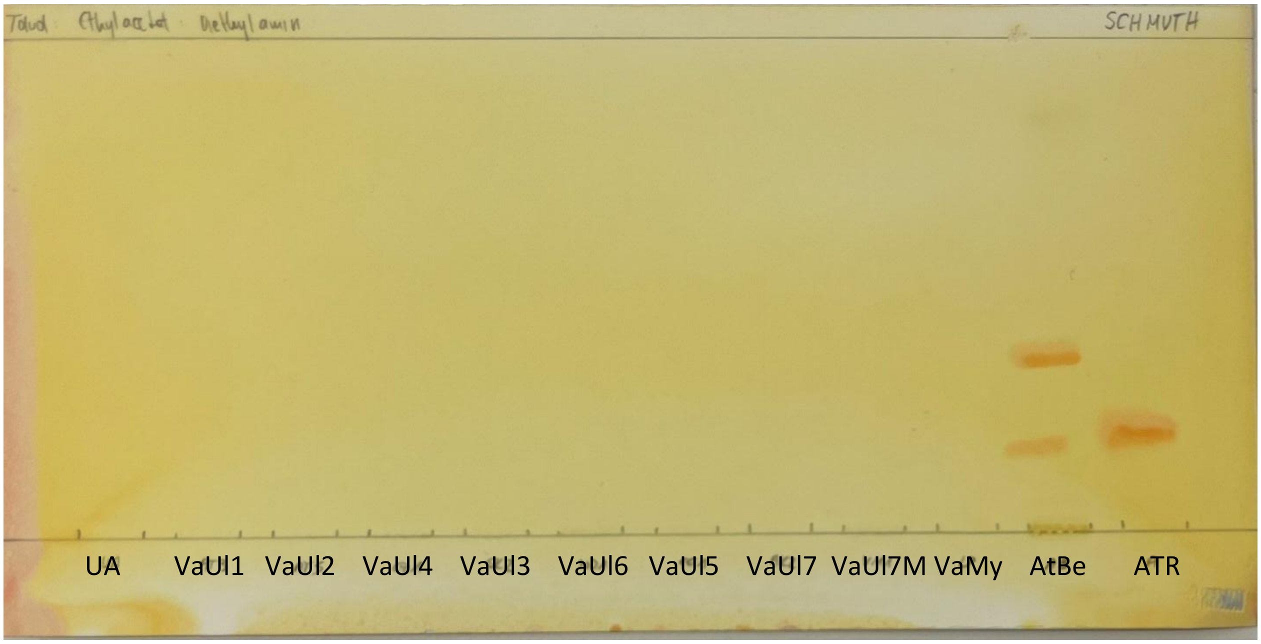

Figure S1: TLC analysis of all Stas-Otto extracts. Mobile phase: Toluol – Ethylacetat – Diethylamin 70:20:10. Stationary phase: TLC silica plate 60 F254. Detection with Dragendorff reagent under visible light. UA – ursolic acid. ATR – atropine.

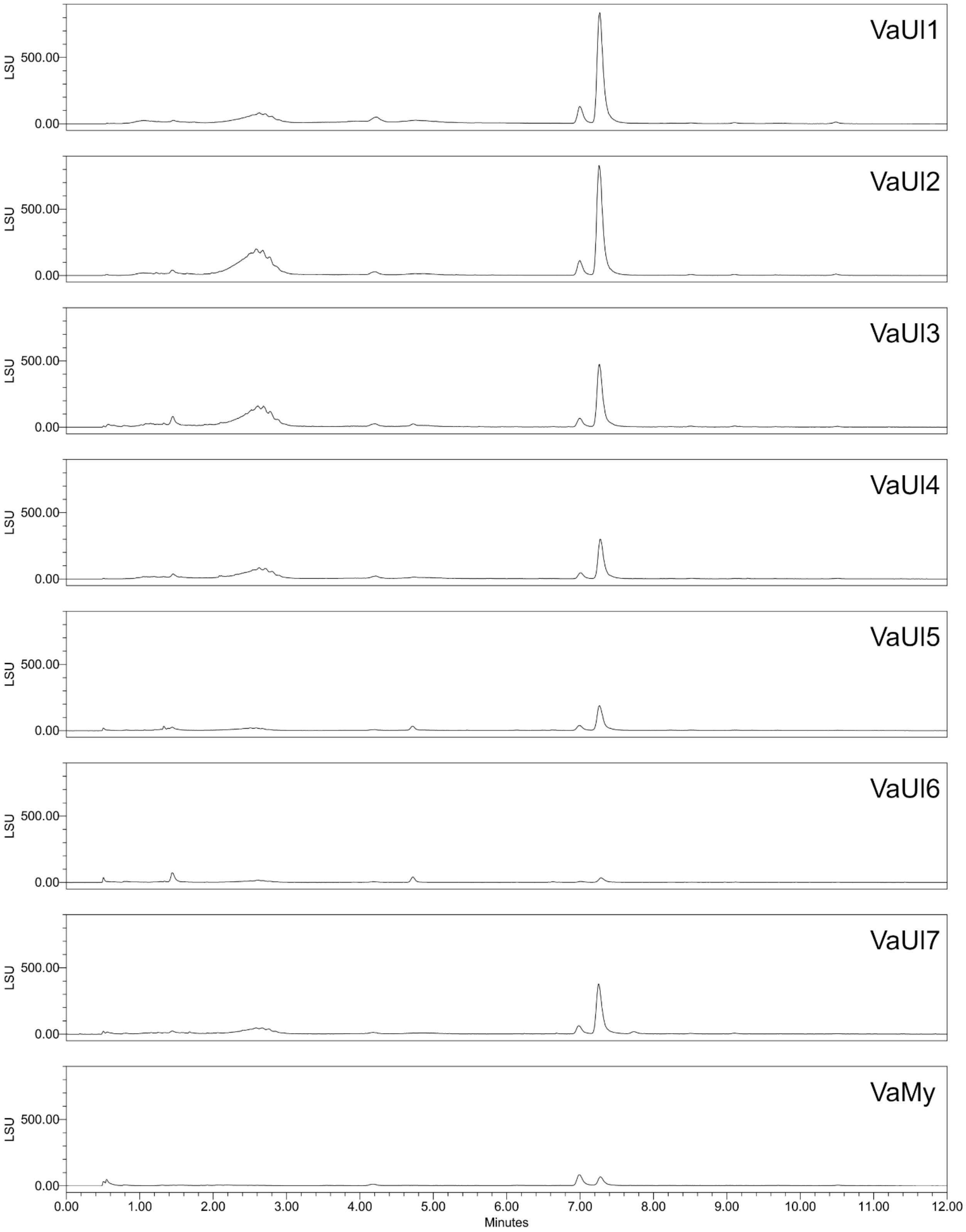

Figure S2: UHPSFC-ELSD traces of all Stas-Otto extracts.

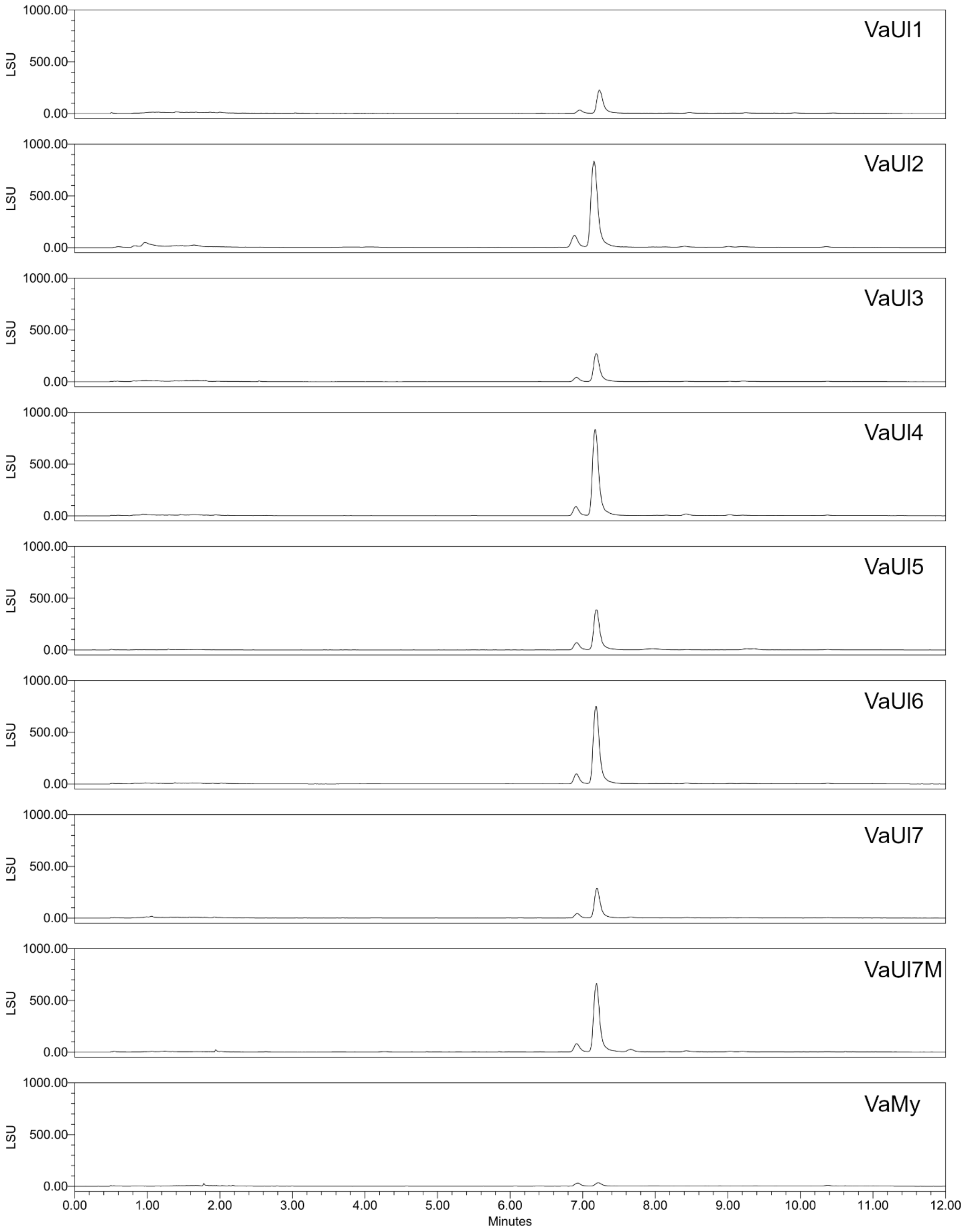

Figure S3: UHPSFC-ELSD traces of all DCM extracts.

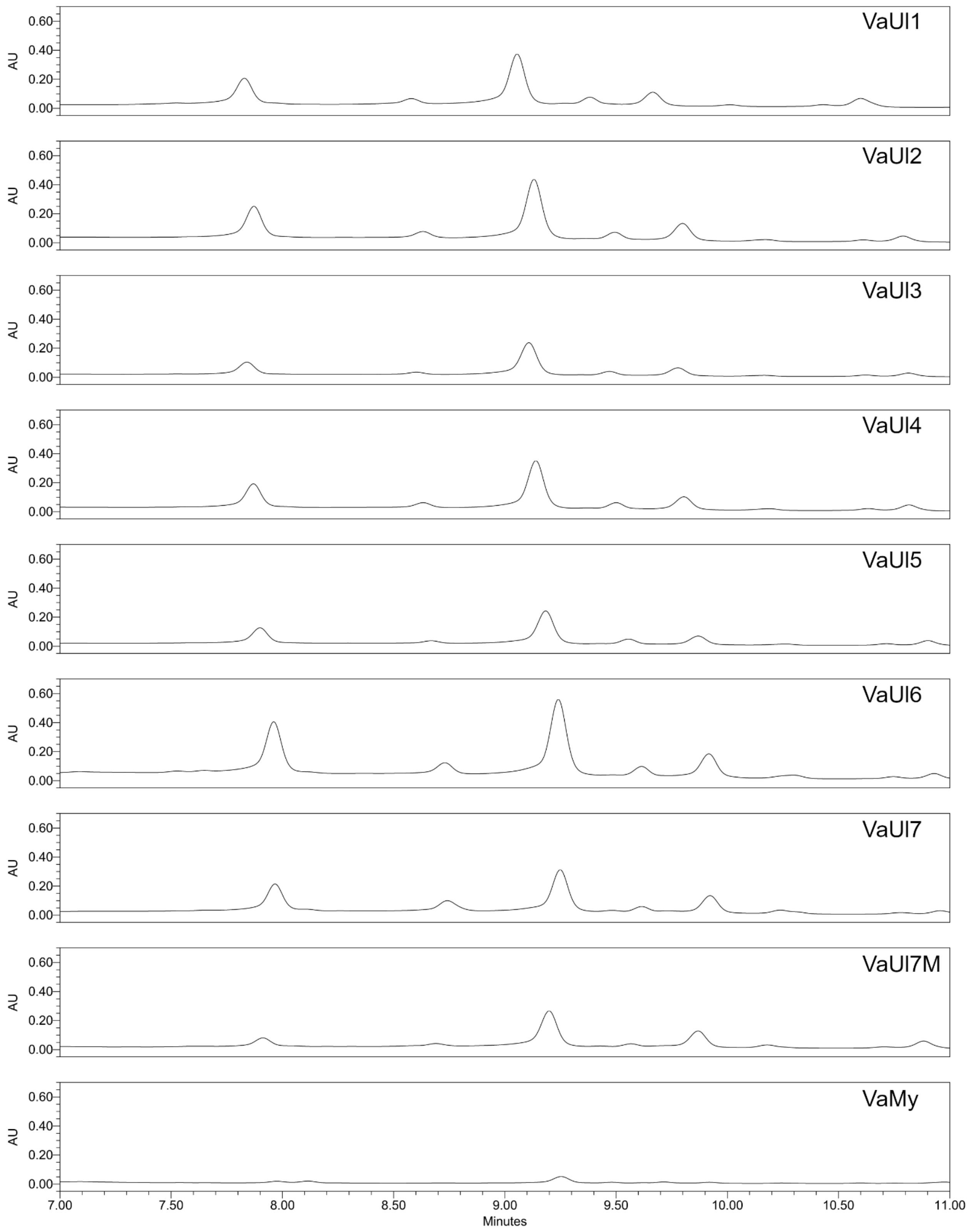

Figure S4: UHPLC-PDA traces of all LLE extracts at 370 nm using System 1

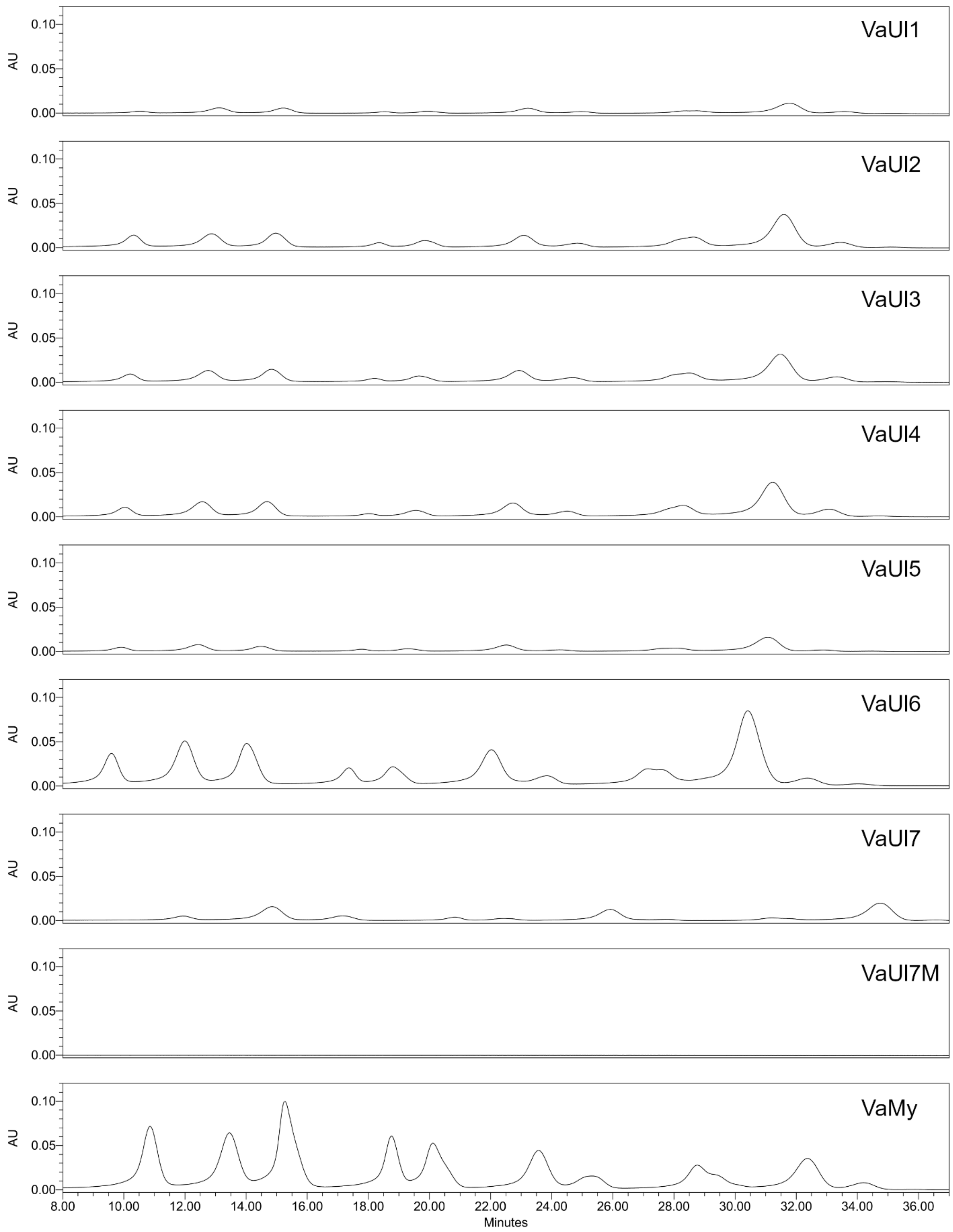

Figure S5: UHPLC-PDA traces of all LLE extracts at 530 nm using System 2

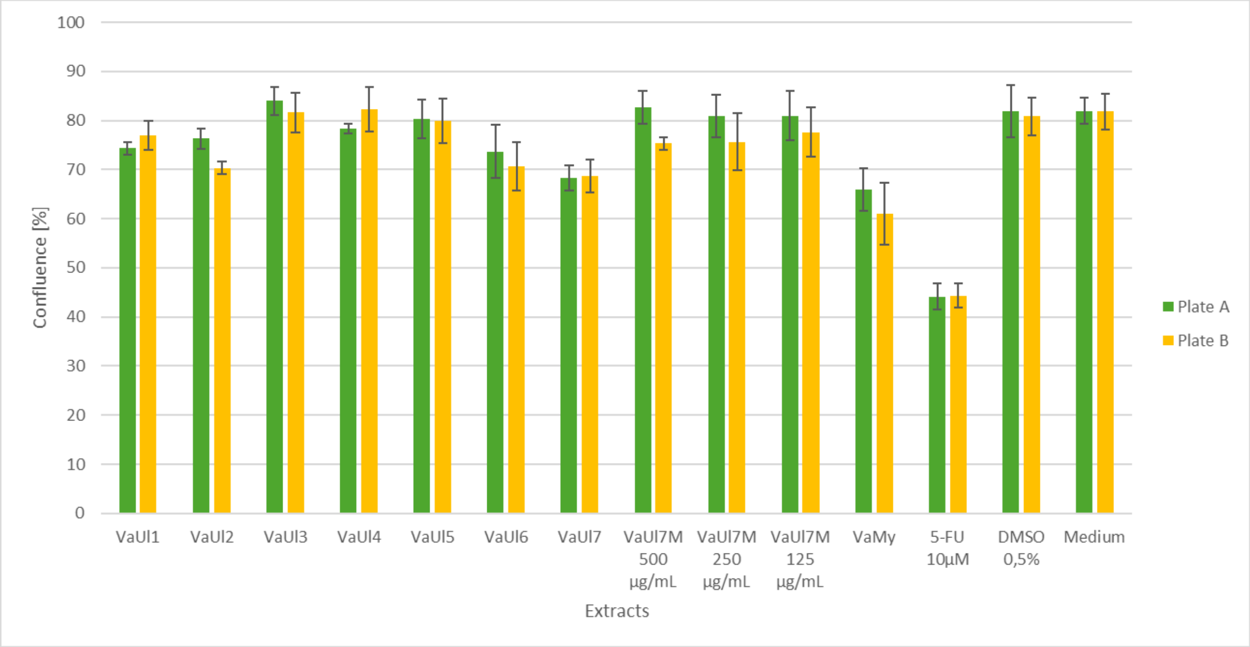

Figure S6: Confluence endpoints of the cell viability assay

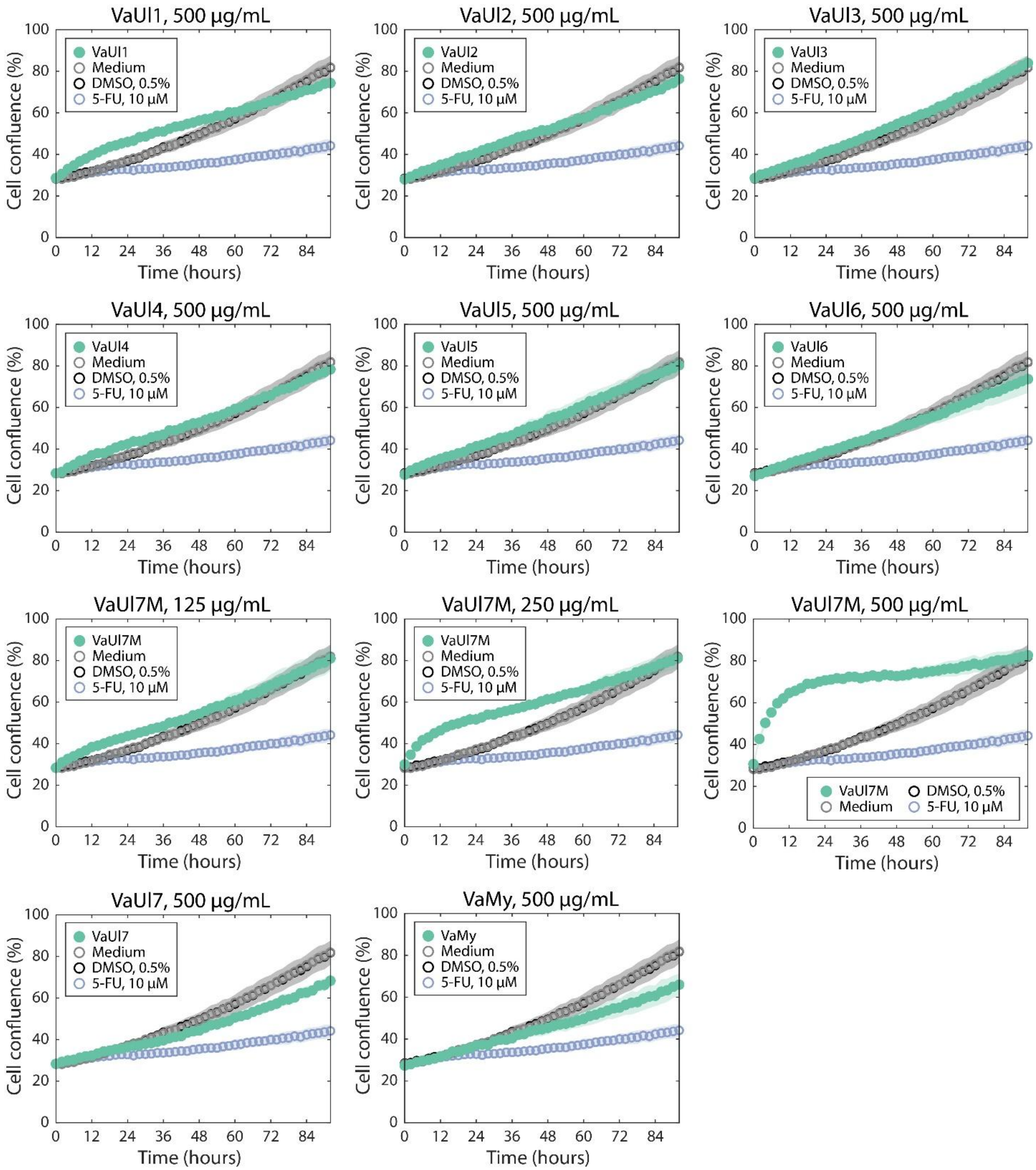

Figure S7: Cell confluence monitoring, showing growth curves for the cells treated with the extracts, compared to the medium and DMSO control, as well as the 5-FU-treated cells.

Table S1: MS annotation of nonpolar extract constituents in the UHPSFC system. Identity of the compounds marked in bold was assigned by comparing the retention time, UV and MS traces with the reference compounds.

Compound	RT [min]	Positive mode *m/z*	Negative mode *m/z*
**β-Sitosterol**	4.10	397.5 $[M–H_2O+H]^+$	457.4 $[M+FA-H]^-$
**Oleanolic acid**	6.95	411.5 $[M-COOH+H]^+$, 439.5 $[M-OH]^+$	455.5 $[M-H]^-$
**Ursolic acid**	7.15	411.5 $[M-COOH+H]^+$, 439.5 $[M-OH]^+$	455.5 $[M-H]^-$
**Maslinic acid**	8.20	409.5 $[M-COOH-OH]^+$, 437.5 $[M-2 OH]^+$, 455.5 $[M-OH]^+$, 473.5 $[M+H]^+$, 495.5 $[M+Na]^+$	471.5 $[M-H]^-$
**Corosolic acid**	8.45	409.5 $[M-COOH-OH]^+$, 437.5 $[M-2 OH]^+$, 455.5 $[M-OH]^+$, 473.5 $[M+H]^+$, 495.5 $[M+Na]^+$	471.5 $[M-H]^-$
**β-Sitosterol glucoside**	10.45	397.5 $[M–H_2O+H]^+$, 599.5 $[M+Na]^+$	621.5 $[M+FA-H]^-$

Table S2: MS annotation of LLE extract constituents in the UHPLC system 1. Identity of the compounds marked in bold was assigned by comparing the retention time, UV and MS traces with the reference compounds.

Compound	RT [min]	Positive mode *m/z*	Negative mode *m/z*
**Myricetin-3-O-galactoside**	7.9	503.1 $[M+Na]^+$	319.2 $[M-Hex-H]^-$, 479.2 $[M-H]^-$
Myricetin pentoside	8.7	473.1 $[M+Na]^+$	319.2 $[M-Hex-H]^-$, 449.2 $[M-H]^-$
**Quercetin-3-O-galactoside**	9.2	487.1 $[M+Na]^+$	301.2 $[M-Hex-H]^-$, 463.2 $[M-H]^-$
Laricitrin hexoside	9.6	517.1 $[M+Na]^+$	331.2 $[M-Hex-H]^-$, 493.2 $[M-H]^-$
**Quercetin-3-O-arabinoside**	9.9	457.1 $[M+Na]^+$	301.2 $[M-Hex-H]^-$, 433.2 $[M-H]^-$

Table S3: MS annotation of LLE extract constituents in the UHPLC system 2. Identity of the compounds marked in bold was assigned by comparing the retention time, UV and MS traces with the reference compounds.

Compound	RT [min]	Positive mode *m/z*	Negative mode *m/z*
Delphinidin-3-O-galactoside	12.6	465.3 $[M]^+$	n.d.
**Delphinidin-3-O-glucoside**	15.8	465.3 $[M]^+$	n.d.
Cyanidin-3-O-galactoside	18.0	449.3 $[M]^+$	n.d.
Delphinidin-3-O-arabinoside	18.5	435.3 $[M]^+$	n.d.
**Cyanidin-3-O-glucoside**	22.0	449.3 $[M]^+$	n.d.
Cyanidin-3-O-arabinoside	23.5	419.3 $[M]^+$	n.d.
Petunidin-3-O-galactoside	24.3	479.3 $[M]^+$	n.d.
**Petunidin-3-O-glucoside**	27.7	479.3 $[M]^+$	n.d.
Peonidin-3-O-galactoside	29.5	463.3 $[M]^+$	n.d.
Petunidin-3-O-arabinoside	30.0	449.3 $[M]^+$	n.d.
**Peonidin-3-O-glucoside**	33.5	463.3 $[M]^+$	n.d.
Malvidin-3-O-galactoside	34.1	493.3 $[M]^+$	n.d.
Peonidin-3-O-arabinoside	34.3	433.3 $[M]^+$	n.d.
**Malvidin-3-O-glucoside**	37.0	493.3 $[M]^+$	n.d.
Malvidin-3-O-arabinoside	39.0	463.3 $[M]^+$	n.d.

Table S4: extraction yields for the three methods used in the study

Sample	Stas-Otto extraction yield [mg/g DW]	DCM extract yield [mg/g DW]	LLE extract yield [mg/g DW]
VaUl1	13,17	37,07	752,1
VaUl2	7,94	18,02	617,5
VaUl3	4,31	21,69	623,6
VaUl4	2,75	13,89	710,8
VaUl5	1,24	12,83	576,9
VaUl6	1,13	13,98	498,2
VaUl7	2,66	12,21	686,6
VaUl7M	2,85	14,17	304,9
VaMy	2,71	10,82	605,7
AtBe	7,18	-	-
